# MDFF_NM: Improved Molecular Dynamics Flexible Fitting into Cryo-EM Density Maps with a Multireplica Normal Mode-Based Search

**DOI:** 10.1021/acs.jcim.3c02007

**Published:** 2024-06-22

**Authors:** Zakaria L. Dahmani, Ana Ligia Scott, Catherine Vénien-Bryan, David Perahia, Mauricio G.S Costa

**Affiliations:** † School of Medicine, Department of Computational and Systems Biology, University of Pittsburgh, 800 Murdoch I Bldg, 3420 Forbes Avenue, Pittsburgh, Pennsylvania 15260, United States; ‡ UMR 7590, CNRS, Museum National d’Histoire Naturelle, Institut de Minéralogie, Physique des Matériaux et Cosmochimie, IMPMC, Sorbonne Université, 4 place Jussieu, Paris 75005, France; § CMCC, Computational Biophysics and Biology, Universidade Federal do ABC, Avenida dos Estados 5001, São Paulo, Santo André 09210-580, Brazil; ∥ Université de Strasbourg—IGBMC—Departament de Biologie structurale integrative, 1 rue Laurent Fries BP, Illkirch 10142 67404, CEDEX, France; ⊥ Laboratoire de Biologie et Pharmacologie Appliquée, UMR 8113, École Normale Supérieure Paris-Saclay, Gif-sur-Yvette 91190, France; # Programa de Computação Científica, Vice-Presidência de Educação, Informação e Comunicação, Fundação Oswaldo Cruz, Av.Brasil 4365, Residência Oficial, Manguinhos, Rio de Janeiro 21040-900, Brazil

## Abstract

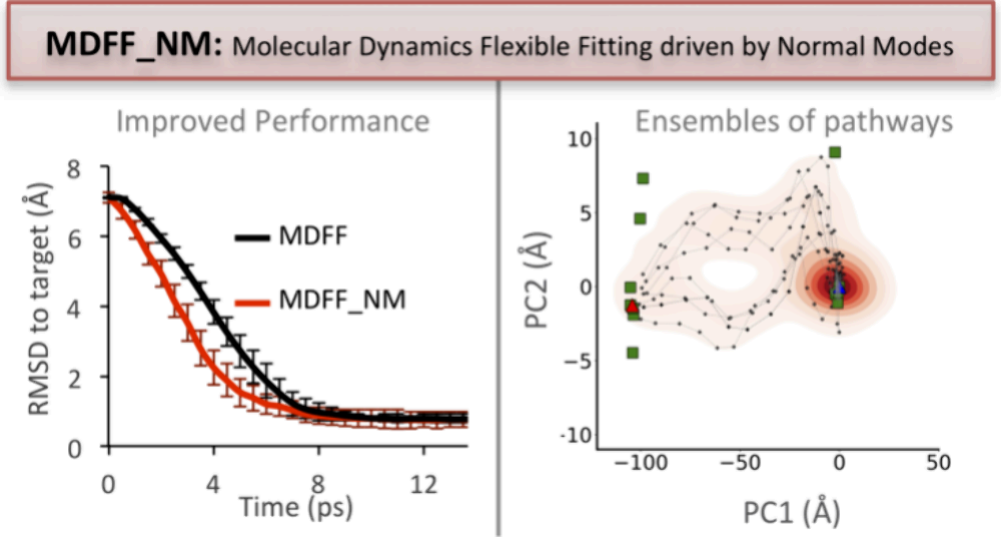

Molecular Dynamics Flexible Fitting (MDFF) is a widely used tool to refine high-resolution structures into cryo-EM density maps. Despite many successful applications, MDFF is still limited by its high computational cost, overfitting, accuracy, and performance issues due to entrapment within wrong local minima. Modern ensemble-based MDFF tools have generated promising results in the past decade. In line with these studies, we present MDFF_NM, a stochastic hybrid flexible fitting algorithm combining Normal Mode Analysis (NMA) and simulation-based flexible fitting. Initial tests reveal that, besides accelerating the fitting process, MDFF_NM increases the diversity of fitting routes leading to the target, uncovering ensembles of conformations in closer agreement with experimental data. The potential integration of MDFF_NM with other existing methods and integrative modeling pipelines is also discussed.

## Introduction

Recent advances in cryo-electron microscopy (cryo-EM) have ignited a revolution in the structural biology community, allowing the resolution of the 3D structure of macromolecular assemblies formed by many interacting components at the atomic level.^1^ Furthermore, Cryo-EM single-particle reconstruction is sometimes the only source of structural information for macromolecular assemblies (e.g., membrane proteins).^2,3^ Current data from the Electron Microscopy Database (EMDB) reveal that 51% of the deposited cryo-EM maps have a resolution above 4 Å.^4^ In such cases, using tools adapted from X-ray crystallography^5,6^ to place atoms into EM densities correctly is not feasible. Rigid-body fitting is a common approach to work with these maps, which can be described as docking the entire structure (or a separate component) onto the EM density.^7^ These search algorithms focus on overall translations and rotations of the macromolecule without considering its internal conformational flexibility. Undoubtedly, rigid-body fitting is a firmly established methodology with many freely available software packages.^8^

In many applications, rigid-body fitting is used as a first attempt to obtain an initial pose that can be further refined with more sophisticated approaches, where the macromolecule’s internal flexibility is considered. Indeed, flexible fitting methods are iterative procedures designed to achieve optimal fitting using successive structural deformations.^9^ In overall terms, we can separate flexible fitting methods into three classes, depending on the search algorithms employed: (i) analytical Normal Mode Analysis (NMA), (ii) molecular simulations, and (iii) hybrid methods combining simulations and analytical calculations.

NMA-based fitting is based on the successive cycles of deformations along selected modes of motion that drive the system toward states with improved fitting.^10−14^ Among flexible fitting approaches, NMA-based methods exhibit the best computational performances, which rely on the fact that accuracy is preserved even with simplified representations, such as elastic network models.^15,16^ Molecular simulations-based fitting usually includes an effective potential derived from Cryo-EM densities, therefore biasing the conformational sampling to conformers closer to the target map.^9,17,18^ From the vast number of available approaches, Molecular Dynamics Flexible Fitting (MDFF) stands out as a popular tool successfully employed to refine many biological systems.^19,20^

When used separately, each of the previously mentioned approaches has significant limitations. NMA-based fitting neglects fast degrees of freedom, which decreases the fitting accuracy in resolutions below 6 Å.^11,12^ Obtaining all-atom models from residue-wise elastic networks is also challenging, potentially leading to the misplacement of side chain atoms.^11,14^ Besides, molecular simulation-based fitting has performance limitations and may require supercomputing facilities to deal with large systems. On the other hand, methods based on NMA may be carried out in single workstations. Regarding accuracy, the standard MDFF approach (also known as direct MDFF) performs poorly in both low (>15 Å) and sub-5 Å resolutions.^21,22^ Standard MDFF was shown to yield distorted unphysical conformations when fitting into poorly defined regions.^21^ Moreover, MDFF refinement at high resolutions exhibits a considerable risk of generating incorrect solutions because of entrapment in the wrong energy minima.^22^

Hybrid methods combining MD and NMA-based fitting are tools with solid potential to overcome the limitations mentioned above.^23,24^ The risk of overfitting commonly related to MD-based flexible fitting^21,22^ is decreased by sampling normal modes directions describing intrinsic motions. In addition, the simultaneous inclusion of fast degrees of freedom leads to a better placement of local elements, improving a fundamental limitation of NM-based methods.^11^ Hence, the available techniques generate solutions with high structural quality at low computational expense.^23,24^

Our group recently developed the hybrid flexible fitting tool MDeNM-EMfit^24^ based on the enhanced sampling method Molecular Dynamics with Excited Normal Modes (MDeNM).^25,26^ According to the procedure, sampling is “guided” along intrinsic motions described by NM directions that maximize the correlation with the experimental map. Apart from other MD-based tools, no bias potential is used during the fitting process. The first reported tests revealed that simulations in the picosecond time scale were sufficient for successfully fitting structures into EM maps of distinct resolutions. However, MDeNM-EMfit outcomes presented slightly higher RMSD values than other MD-based flexible fitting methods. This observation may be due to the lack of a biasing potential guiding the simulations.^24^

We present an optimized approach to flexible fitting by combining the strengths of MDeNM-EMfit with MDFF, the latter being widely recognized as the most used MD-based fitting method.^20^ In other words, this proof-of-concept study aims to evaluate the impact of including a conformational search guided by normal modes on MDFF. Different parameters associated with the fitting process were assessed to provide a comparative view of the method’s accuracy, performance, and usefulness for generating conformational ensembles in agreement with the Cryo-EM map. These ensembles are likely to capture the local variability from a discrete number of conformations, opening the door for assessing the conformational heterogeneity, a richer palette of analysis on the ensemble data, and multiple transition paths to be explored.

## Theory

### Combining MDFF and MDeNM-EMfit

Standard MDFF uses a biasing potential that adds forces proportional to the gradient of the density map. This biasing potential guides the atomic structure toward high-density regions in cryo-EM density maps.^19^ It includes a regular force field-based potential *U*_MD_, a biasing potential *U*_EM_, and a restraining term *U*_SS_ to ensure the stability of the secondary structure contents and decrease the risk of overfitting in the process. Besides these potentials, we designed MDFF_NM to include a conformational sampling along deformations described by NMs that lead the system to conformers exhibiting maximal correlation with the EM map. This involves successive kinetic excitations of combinations of NMs, scaled according to a factor that controls the temperature increase during short MD simulations.

**Figure 1:**
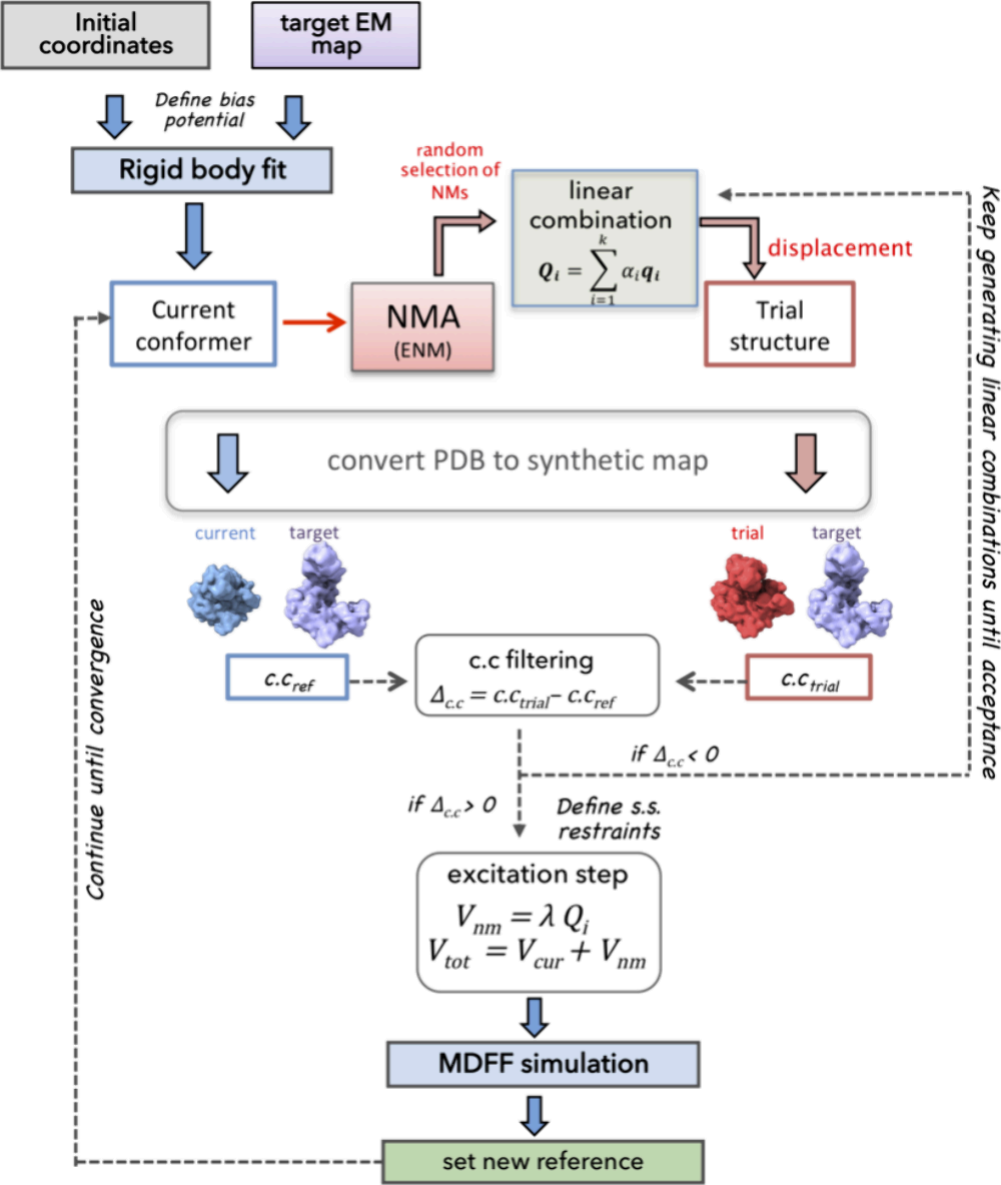
Schematic summary of the MDFF_NM algorithm for a single replica.

MDFF_NM naturally emerged as an optimization of MDeNM-EMfit^24^ and MDFF,^19^ creating a synergy that draws from the best of both approaches (see Figure 1). The pivotal element lies in the combination of a multireplica approach allowing the exploration of structure-encoded motions leading to the target state (provided by MDeNM-EMfit) and the influence of the *U*_EM_ biasing potential that guides the exploration (provided by MDFF). Consequently, simulations are anticipated to trace relaxed pathways, which require weaker biasing forces to fit the structures into maps.

## Algorithm

MDFF_NM is a multireplica approach where M-independent simulations can run in parallel or sequentially. Each replica contributes by generating a transition path and final conformer. Therefore, beyond its primary function as a flexible fitting procedure, MDFF_NM also provides a conformational ensemble approach, following these steps:

1. Identify an electron density map for a given protein (target map—TM) for which one would like to fit a 3D atomic structure.
2. Select an atomic structure for the same protein in a different conformation. A theoretical model can be used here (we will call it starting structure (S1)).
3. Perform rigid-body fitting of S1 in TM.
4. Start M processes corresponding to the number of replicas (and ultimately the number of fitted structures) one wants to generate.
5. Generate a synthetic map for S1 and calculate the cross-correlation (*c.c*) with the TM. Then, store this value as *c*.*c*_ref_.
6. NMA step: for each replica, build an atomistic elastic network model (ENM) for S_*i*_ (structure corresponding to the *i*^th^ iteration) and compute the NMs.
7. Selection of NMs: for each replica, randomly pick *k* (with *k* being a user-defined value) lowest frequency NMs according to the following probability distribution, as described in ref (27):
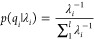
1where *p*(*q*_*i*_|λ_*i*_) is the probability of picking mode *q*_*i*_ given its eigenvalue λ_*i*_. According to previous studies in this field, combinations of three modes enable a proper exploration of conformational spaces.^28^ Thus, this stochastic mode selection process enables replicas to take different paths.
8. Linear combinations: for each replica, randomly scale each selected mode using a scalar drawn from the interval α∈[−0.5,0.5] with a uniform probability distribution. Then, linearly combine the selected modes to form a displacement vector ***Q***:
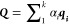
2
9. Testing combination of NMs: displace S_*i*–1_ along ***Q*** up to produce a trial S_*i*_ structure (t-Si). Generate a synthetic map from this structure and calculate its cross-correlation with TM (*c.c*_trial_). Then, calculate the difference in cross-correlation (Δ*c*.*c*) between *c*.*c*_ref_ and *c.c*_trial_ as follows:
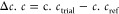
3If Δ*c*.*c* is higher than zero, the combination is accepted, otherwise return to step 7 for selection of other modes.
10. Excitation step: compute the mass-weighted kinetic excitation factor λ and generate a scaled new vector ***Q***_**exct**_. Add this vector to the current velocities from the previous iteration (*v*_curr_), or if it is the first iteration, use velocities from the last frame of equilibration:
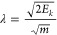
4
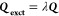
5
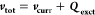
6
11. Perform MDFF simulation using structure S_*i*_ and velocities *v*_tot_.
12. Generate a synthetic map from the last frame of the simulation, then compute the *c.c* with the TM and set it to *c*.*c*_ref_.
13. Repeat from step 6 until convergence.

## Results and Discussion

Three distinct test systems in Table 1 were considered to compare standard MDFF and MDFF_NM. Each system underwent 10 independent runs of either MDFF or MDFF_NM using the same starting structures and target maps. The definitions for the MDFF bias potential, secondary structure, and chirality restraints were the same for both approaches (details in the Methods section), therefore enabling a direct evaluation of the inclusion of NM-based exploration. Three descriptors were adopted in the assessment of both methods: (i) the *c.c* with the EM map, (ii) the Root Mean Square Deviation (RMSD) with respect to the structure considered to produce the target maps, and (iii) the molprobity score,^29^ providing an estimation of the overall structural quality of the generated conformers.

**Table 1:** Structural Data Used in This Study

**system**	**initial PDB**	**target PDB**	**initial RMSD (Å)**
adenylate Kinase	1AKE(30)	4AKE(31)	7.1
LAO *binding* protein	2LAO(32)	1LST(32)	4.6
GroEL monomer	1SX4(33)	2C7C(34)	11.7

### Evaluating the Accuracy and Performance of MDFF_NM

**Figure 2:**
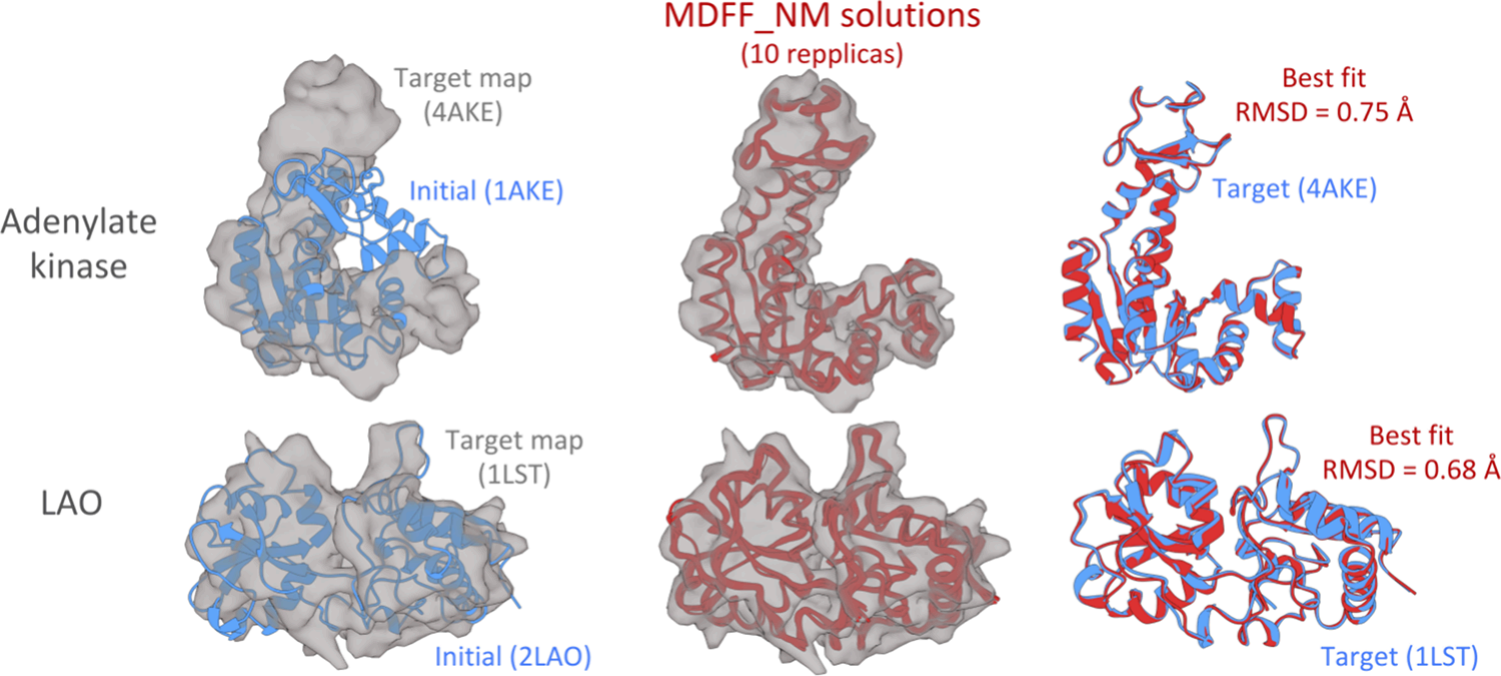
Flexible fitting results obtained with MDFF_NM. Left: cartoon representation of the initial structures (blue) with the corresponding target maps in 5 Å resolution (gray). Center: superposition of the 10 best solutions (red) into their target maps. Right: structural alignment between the best-fit conformer (red) and the target structures (blue).

We first assessed the accuracy of MDFF_NM. Figure 2 illustrates the aligned conformational ensembles obtained for Adenylate Kinase (ADK) and LAO binding protein docked within their respective density maps (center) and the solutions exhibiting the highest *c.c* aligned to their target experimental structures (right). MDFF_NM outputs fit correctly into the map, as evidenced by the low RMSD values with respect to the structures. The ensembles obtained for both proteins exhibit variations at flexible loops in agreement with previous studies.^24,27^

**Figure 3:**
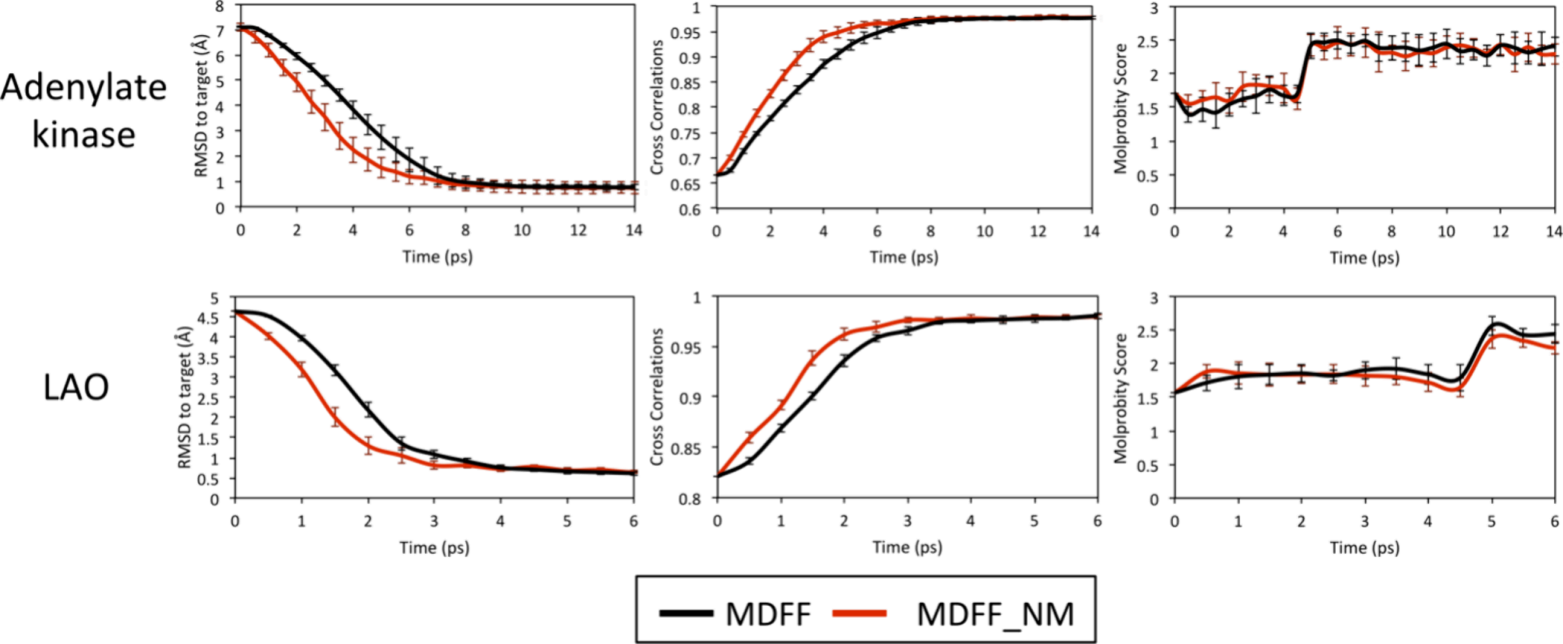
Performance evaluation of MDFF_NM. Each considered test case is indicated on the left of the plots. Time evolution of the average RMSD taking as references the structures considered for generating the target maps (left), cross-correlation coefficients (center), and molprobity scores (right). Average values were computed from 10 independent simulations performed in vacuum.

Next, we investigated the convergence time of each method as illustrated in Figure 3. Notably, MDFF_NM demonstrated faster convergence than MDFF. In the RMSD plots, slightly larger error bars were observed for MDFF_NM in the first cycles due to the added energy along normal modes. However, their magnitudes were around 0.5 Å, which indicates no significant structural perturbations along the pathway. The molprobity score panel revealed no significant differences between the two methods. The increase in molprobity scores during the fitting simulations is in line with values obtained for the target states considered for generating EM maps (Supplementary Table 1).

MDFF_NM also outperformed MDeNM-EMfit in terms of both the accuracy of the solutions and the number of steps required to reach them (Supplementary Figure 1). This result could be attributed to the incorporation of synergistic effects present in MDeNM-EMfit and MDFF. In summary, MDFF_NM exhibits improved performance while maintaining fitting accuracy and the ability to generate intermediate structures and final solutions of comparable quality to standard MDFF.

### Testing MDFF_NM in Distinct Conditions

**Figure 4:**
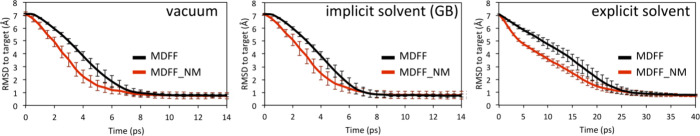
Testing MDFF_NM performance under different solvent representations. Average RMSD with respect to the target Adenylate kinase structure considered for generating the 5 Å synthetic map (4AKE.pdb). For each evaluated approach, 10 independent simulations were performed. Colored as in Figure 3.

One significant advantage of MDFF_NM is the possibility of working with different complexity levels. As pointed out before, in some cases, the inclusion of explicit solvent in MDFF simulations impacts the quality of the results.^35^ Thus, we also investigated the convergence times for both methods in the presence/absence of solvent (Figure 4). In all cases, MDFF_NM outperformed the standard procedure. Regarding accuracy, sub 1 Å solutions were achieved in all solvent representations, and including implicit solvent had a minimal impact on the required MD steps to reach convergence. As expected, explicit solvent simulations required more steps to converge.

### Fine-Tuning of Parameters

As pointed out, MDFF parameters were chosen following previous studies.^19,20,36^ Here, we evaluated the influence of two key parameters related to the exploration of the normal modes space: the percentage of low-frequency modes considered for building linear combinations and the excitation energies (kinetic energy injected along selected NM combinations). As shown in Supplementary Figure 2, variations in the percentage of normal modes had a minimal effect. On the other hand, the magnitude of the injected excitation energies severely impacted both performance and accuracy (Supplementary Figure 3). While excitation energies in the 10 to 20 kcal/mol range already improved the performance compared to standard MDFF, simulations using Ek ≥50 kcal/mol exhibited substantial gain in performance. However, there was a noticeable loss of accuracy in simulations using Ek = 100 kcal/mol, as given by the large error bars and the detrimental quality (higher RMSD values) compared to simulations using Ek = 50 kcal/mol.

### Escaping Energy Minima with MDFF_NM

**Figure 5:**
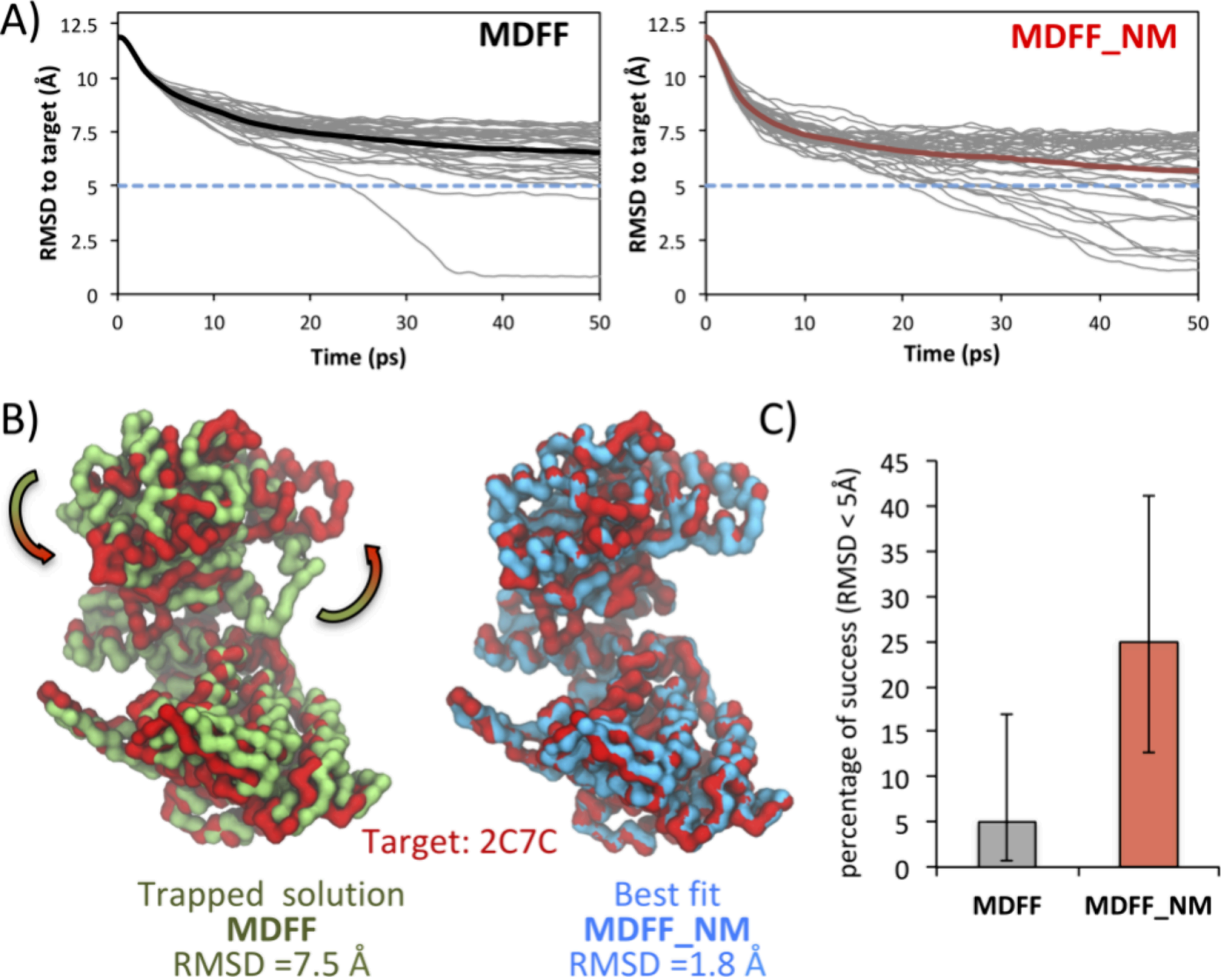
Escaping GroEL energy minima with MDFF_NM. (A) RMSD as a function of time for a set of 40 replicas per method (gray curves). The average RMSD values a represented in black and red for MDFF and MDFF, respectively. (B) Superposition of representative solutions onto the target structure (red). The trapped state obtained in MDFF simulations is green, and the correct fitting obtained with MDFF_NM is represented in blue. The arrows indicate the motion required to reach the target state. (C) Results from an exact binomial test. A final RMSD below 5 Å defined a successful run. The bars indicate the 95% confidence interval.

We also applied both methods to the flexible fitting of the GroEL monomer, which is considered a challenging system, as given by the ∼12 Å RMSD difference between the initial and target structure. Given that our initial set of 10 standard MDFF simulations did not generate any solution exhibiting a RMSD below 5 Å with the target structure, we included 30 additional replicas, totaling 40 independent runs. As shown in Figure 5A, most solutions were around 7.5 Å RMSD, while two reached a sub-5 Å threshold. To enable fair comparisons, we performed the same number of MDFF_NM replicas. The resulting plot shows that the average RMSD value was below the one obtained with the standard method, and a more substantial number of replicas reached the sub-5 Å threshold. Using this criterion to evaluate the fitting success, we demonstrate that MDFF_NM statistically surpasses the standard method (Figure 5C). Standard MDFF failure is due to the method’s difficulty dealing with domain rotations,^21^ as exemplified by the trapped state obtained for most solutions (Figure 5B). To escape this state, a significant rotation of the top part of the structure is required to reach the correct solution. This MDFF_NM improvement might be attributed to the increased ratio of escaping wrong energy minima by excitation of structure-encoded motions usually describing interdomain transitions (Figure 5B).

### MDFF_NM Generates Conformational Ensembles in Closer Agreement with Experimental Data

**Figure 6:**
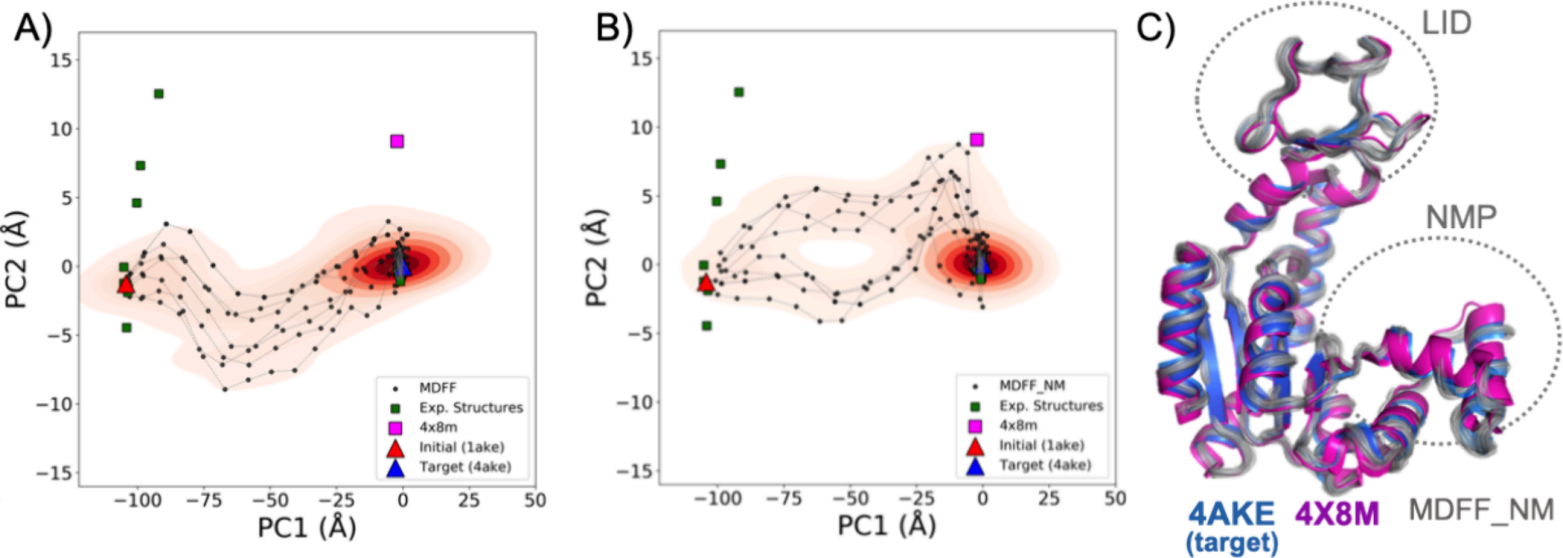
Projection of intermediate structures onto principal components calculated from an ensemble of ADK experimental structures. (A) MDFF results in a unique transition pathway with limited variability. (B) MDFF_NM results exhibit higher pathway variability. A better coverage of the space spanned by experimental structures is also obtained. The contour maps highlight the ensembles of conformers generated during the fitting simulations. (C) Superposition between the target and a distinct open ADK state sampled only in MDFF_NM simulations (4 × 8 M—magenta square), exhibiting structural variations on the LID and NMP domains as indicated. The MDFF_NM-generated snapshots are also provided.

As previously discussed, ensemble-based flexible fitting not only improves the quality of the fitted models but also yields a plausible interpretation of the conformational heterogeneity of the data. We compared the exploration of the conformational space generated by MDFF and MDFF_NM, focusing on the transition pathways connecting the initial and target states (Figure 6). As expected, due to its deterministic nature, there is minimal variation among pathways generated by MDFF. Further, these routes did not cover a region of the conformational space populated by experimental known states (Figure 6A).

On the other hand, pathways obtained by MDFF_NM could be split into two separate groups, likely related to the stochastic nature of the method. Moreover, MDFF_NM-generated pathways explore a region of the conformational space closer to known experimental states than MDFF (Figure 6B). Figure 6C highlights that only MDFF_NM could sample a distinct open conformation (PDB ID: 4X8M) before arriving at the target state. Altogether, these results show that including an NM-driven mechanism into MDFF_NM enables more accurate sampling of the conformational space, which is probably associated with the method’s ability to follow structure-encoded motions adaptively. In contrast, MDFF is known for introducing large forces to deform structures without aiming at obtaining relaxed pathways, which often results in sampling unphysical conformations.^21^

### Room for Improvement

In this study, we used commonly adopted biasing parameters for MDFF while optimizing the NM-related configurations. Further optimizations will be carried out by testing different choices of biasing parameters (e.g., biasing forces controlled by the *gscale* option) and varying magnitudes of secondary structure and chirality restraints. The MDFF_NM performance could also be improved by implementing the NMA subroutines directly into the NAMD/VMD ecosystem. Indeed, the *i*/*o* processing routines by the R software were the main performance bottlenecks.

There are many known limitations related to flexible fitting using standard MDFF or other early developed tools.^21,37^ Besides the high computational cost, they exhibit a great potential for overfitting, and both accuracy and performance can be compromised due to entrapment within local minima. In the past decade, some MDFF-based tools were developed to overcome these limitations.^20,37^ Their rationale is to incorporate computationally generated blurred maps derived from the experimental data, thereby enabling the refinement against a series of maps with progressively higher resolutions.

Both cascade MDFF (cMDFF) and the resolution exchange MDFF (ReMDFF) exhibited improved performance than the standard procedure, particularly when fitting structures into high-resolution maps.^20,37^Supplementary Figure 4 compares MDFF_NM and the cascade MDFF implementation (cMDFF). Although cMDFF was faster than the standard procedure, MDFF_NM still performed best. It should be noted that cMDFF involves a series of successive runs decreasing the degree of blurring of the target map until maximal resolution. As discussed in the original cMDFF article, to achieve maximal performance, users must carefully control the simulation lengths and steps adopted to decrease the level of blurring. Consequently, each simulated system may require different parameters during fitting.^37^ Therefore, the performance of cMDFF could be further enhanced through systematic parameter optimization, although this was beyond the scope of our study.

The combination of the cascade procedure with MDFF_NM holds the potential to leverage advantages from both approaches. The gradual decrease of blurring combined with a conformational search along intrinsic motions could accelerate the fitting process. This integrated implementation will be the focus of future studies from our group. We also intend to incorporate specific routines to enhance the fitting of local elements, as observed in the Correlation-Driven Molecular Dynamics (CDMD) method. In this tool, after convergence of the MD-based fitting, a high-temperature-simulated annealing is applied to improve the placement of side chain rotamers.^38^

Furthermore, the MDFF_NM approach presented here could be straightforwardly adapted to incorporate certain features from the ReMDFF resolution exchange approach. As both methods are replica-based, the NM-driven conformational search could also be conducted concurrently in blurred maps of varying resolutions. Enhanced performance may also be achieved by combining MDFF_NM with the recently developed R-MDFF approach (RADICAL augmented MDFF). The latter is an ensemble-based flexible fitting tool that also demonstrated encouraging results.^39^ Once again, given that both MDFF_NM and R-MDFF are replica-based tools, incorporating NM-driven sampling into the latter would not disrupt the inherent nature of the method.

## Conclusions

MDFF_NM is a stochastic hybrid flexible fitting algorithm combining NMA and MD-based flexible fitting that also enables the massive exploration of portions of conformational landscapes defined by the Cryo-EM density maps. In addition to speeding up the fitting process, MDFF_NM generates alternative fitting routes by introducing NMA-based stochastic excitations, uncovering novel previously inaccessible pathways through MD-based flexible fitting. Furthermore, the procedure is conducted considering the dynamics of fast degrees of freedom during atomic resolution simulations, thus overcoming a significant limitation of NMA-based flexible fitting.^11,40^ Finally, combined with a multireplica scheme, the stochastic component increases the potential of obtaining a correct fit compared to traditional deterministic methods. Although MDFF_NM was designed as a highly parallelizable method with default parameters suitable for most applications, expert users can tune the protocol to extract optimal results for their specific cases.

In light of the recent technical and methodological advances in cryo-EM, the analysis of conformational heterogeneity from experimental particles stands out.^41,42^ Rather than focusing only on obtaining high-resolution structures, many groups are developing methods devoted to a more comprehensive exploration of conformational landscapes. Through these methods, considerable growth is expected in the number of generated maps with medium resolution, thereby requiring fast and accurate ensemble-based fitting approaches to properly deal with large pieces of data.^40,43^ In this context, significant advances in flexible fitting into midresolution maps were obtained by integrative approaches, such as the CryoFold tool that integrates ReMDFF into MELD (Modeling Employing Limited Data).^44,45^ Given the potential for generating of ensembles and the computational performance demonstrated by MDFF_NM, future perspectives include integrating the method into this platform or in other tools, such as the Metainference framework.^46^ Finally, a key point of interest is the direct implementation of MDFF_NM into the NAMD/VMD ecosystem, thereby contributing to the utilization of this method by a large community of researchers.

## Methods

### Atomic Coordinates and Experimental Data Set

The atomic coordinates selected for the calculations performed in this study were downloaded from the Protein Data Bank.^47^ They are listed in Table 1 together with the initial RMSD between the initial and target states. The ensemble of ADK experimental conformations was obtained using ProDy v.1.9.3.^48^ First, we carried out a blast search against the PDB to retrieve structures sharing at least 90% sequence identity with the reference structure 1AKE.^30^ A list of the PDB IDs of this experimental data set is provided in the supporting material. Then, the *C*_α_ atoms from these structures were superposed using the Kabsch algorithm. The most relevant structural variations found in the data set were identified with a principal component analysis (PCA). Briefly, PCA is based on the diagonalization of the covariance matrix, *C*_(i,j)_, of atomic positions whose elements are represented by
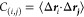
7where Δ*r*_*i*_ and Δ*r*_*j*_ indicate the displacement vectors of atoms *i* and *j*, respectively, from their average positions, brackets indicate ensemble averages. Then, an eigenvalue problem is solved, resulting in 3N PCs that can be sorted according to their fractional contributions to the overall variance. In our case, the cumulative contribution provided by the first two PCs was approximately 95%.

### Preparation Steps and Generation of Simulated Maps

The generation of simulated maps from target structures (Table 1) followed the steps described in the MDFF tutorial provided in (https://www.ks.uiuc.edu/Training/Tutorials/science/mdff/tutorial_mdff-html/). Briefly, this procedure required the *psfgen* and *mdff* plugins included in the visualization software VMD 1.9.4.^49^ While 5 Å resolution maps were generated for the ADK and LAO proteins, for GroEL, a 8.7 Å resolution map was generated in accordance with the resolution found in the reference Cryo-EM structure (EMDB 1181). The rigid-body docking of the initial structures into the generated densities was performed using the voltool fit command in VMD. Secondary structure and chirality restraints were defined using the default parameters provided in the tutorial.

### Equilibration of Starting Structures

All simulations were carried out using NAMD v. 2.14^50^ with the CHARMM 36m force field.^51^ Simulations were performed in vacuum. Nonbonded interactions were calculated up to 10 Å, approximated until 12 Å by using a force-switching function. Initially, systems were energy-minimized, starting with the conjugate-gradient algorithm, keeping protein heavy atoms harmonically restrained with a force constant of 10 kcal mol^–1^ Å^–2^ to avoid structural distortions. Then, the atomic velocities were assigned accordingly to a Maxwell–Boltzmann distribution corresponding to 50 K and then slowly increased to 300 K during a 1 ns heating MD using a 1 fs integration time. In the equilibration step, the positional restraints were gradually decreased to zero during the first half of a 2 ns constant temperature MD using the Langevin thermostat with a damping coefficient of 1 ps^–1^. All restraints were removed in the remaining part of the equilibration.

### MDFF Simulations

MDFF simulations were performed in vacuum, except for the ADK system where simulations were also carried out in different distinct degrees of complexity: implicit solvent using the GBIS model^52^ or explicit solvent under periodic boundary conditions. The biasing forces were applied to nonhydrogen atoms using a grid force-scaling factor of 0.3 (*gscale* option). The atomic mass of each selected atom was used for the atom-dependent weight. Simulation parameters were kept unchanged from the equilibration procedure. The temperature was kept constant at 300 K. The MD integration time adopted for production simulations was 1 fs.

For the implicit solvent simulations, the cutoff for Born radius calculations was 14 Å and nonbonded interactions were calculated up to 15 Å, being approximated until 16 Å by a force-switching function. The simulated concentration of ions was 0.1 M. Solvent dielectric was set to 80. The other GBIS related parameters were left as default. Explicit solvent simulations used the following configurations: nonbonded interactions were calculated up to 10 Å, being approximated until 12 Å by using a switching function. Electrostatic interactions were treated with the PME algorithm using a 10 Å cutoff.^53^ The SETTLE^54^ and SHAKE^55^ algorithms were used during MD simulations to fix bonds involving hydrogen atoms in water molecules and protein. Pressure was kept constant at 1 atm during equilibration and production using the Langevin piston method.^56^ In these steps, temperature was kept constant at 300 K using the Langevin thermostat with a damping coefficient of 1 ps^–1^.

### MDFF_NM Simulations

All simulations were carried out following the procedure presented in Figure 1. The MD simulation steps were performed with NAMD using the same configurations adopted for standard MDFF simulations. The *c.c* calculations on intermediate structures were performed with the *voltool* command in VMD. The overall management of files, normal mode calculations, and *i*/*o* processing were performed with R v.3.5.1^57^ in conjunction with the Bio3D package v. 2.3.3.^58,59^ Normal modes calculations were performed using the AAENM atomistic elastic network model^60^ with the rotation-translation blocks (RTB) approximation^61^ (one residue per block). Different percentages of modes defining the relevant subspace were tested in our simulations without a major impact on the results (Figure S2). Therefore, only 1% of the lowest frequency modes calculated at the beginning of each cycle were considered to search for excitation directions. Unless otherwise stated, excitation energies were set to 50 kcal/mol. The procedure converges when no increase in *c.c* is observed after 2 consecutive cycles.

### MDeNM-EMfit Simulations

All simulations followed the protocol described in our previous paper.^24^ The LAO binding protein starting structure and target state were the same as those selected for MDFF simulations (Table 1). Briefly, Simulated maps were generated with the EMAN2 software^62^ using the pdb 2mrc routine. Fitting of atomic structures into the reference maps and *c.c* calculations were performed with the fitmap module of Chimera.^63^ MDeNM-EMfit simulations were conducted using R v.3.5.1^57^ in conjunction with the Bio3D package v.2.3.3.^58,59^ Normal modes calculations were also carried out using the AAENM model included in the Bio3D package.^60^ The MD simulation steps were performed in vacuum using CHARMM v.42b1.^64^ The same force field parameters adopted for MDFF simulations were also used here, allowing a direct comparison of the results. Excitation energies were set to 50 kcal/mol applied each picosecond.

### Cascade MDFF (cMDFF) Simulations

All simulations were carried out following the protocol described in ref (38). The ADK starting structure and target state were the same as those selected for MDFF simulations (Table 1). Briefly, blurred simulated maps were generated with VMD using a 1 Å resolution step. We also tested a 0.5 Å resolution step, but this configuration resulted in poorer performance. All simulation parameters were kept unchanged from standard MDFF simulations performed in this study. Here, 2 ps simulations were performed at each of five resolutions starting from 9 until 5 Å.

## Data Availability

The MDFF_NM code is provided in the Supporting Information as a compressed zip file. All associated scripts and inputs required for running fitting simulations are also included in the same file.

## Supporting Information Available

(Figure S1) Comparing the performance of MDFF_NM and MDeNM-Emfit, (Figure S2) testing MDFF_NM parameters in flexible fitting: percentage of normal modes, (Figure S3) testing MDFF_NM parameters in flexible fitting: kinetic energy injected, (Figure S4) comparing the performance of different MDFF implementations, and (Table S1) comparison between molprobity scores obtained for MDFF_NM solutions and their corresponding target states (PDF)
PDB identifiers of ADK structures are also provided for comparisons with simulated data and a compressed.zip file containing the MDFF_NM code, scripts, and input files is also provided (ZIP)
