# Supplementary material for: MDFF_NM: Improved Molecular Dynamics Flexible Fitting into Cryo-EM Density Maps with a Multireplica Normal Mode-Based Search
Source: J Chem Inf Model. 2024 Jun 22;64(13):5151–60. doi: 10.1021/acs.jcim.3c02007 (PMC11234365; doi:10.1021/acs.jcim.3c02007)
Supplement: Supplementary file 1 — ci3c02007_si_001.pdf [file ci3c02007_si_001.pdf]

Supporting Information

MDFF_NM: Improved Molecular Dynamics Flexible Fitting into Cryo-EM Density Maps with a Multi-replica Normal Modes-based Search

Zakaria L. Dahmani[1, 2], Ana Ligia Scott[3,4], Catherine Vénien-Bryan[2], David Perahia[5*] & Mauricio G.S Costa*[2,5,6]

[1] University of Pittsburgh, School of Medicine, Department of Computational and Systems Biology, 800 Murdoch I Bldg, 3420 Forbes Avenue, Pittsburgh, PA 15260, USA.

[2] Sorbonne Université, UMR 7590, CNRS, Museum National d'Histoire Naturelle, Institut de Minéralogie, Physique des Matériaux et Cosmochimie, IMPMC. 4 place Jussieu, 75005, Paris, France.

[3] Universidade Federal do ABC, CMCC, Avenida dos Estados 5001, 09210-580, Sanao André- SP., Brazil.

[4] Université de Strasbourg- IGBMC -Departament de Biologie structurale integrative 1 rue Laurent Fries BP 10142 67404 Illkirch CEDEX

[5] Laboratoire de Biologie et Pharmacologie Appliquée, UMR 8113, École Normale Supérieure Paris-Saclay, 91190 Gif-sur-Yvette, France.

[6] Fundacao Oswaldo Cruz, Programa de Computação Científica, Vice-Presidência de Educação, Informação e Comunicação. Av.Brasil 4365, Residência Oficial, Manguinhos. 21040-900, Rio de Janeiro / Brazil.

To whom correspondence should be addressed:

mauricio.costa@fiocruz.br

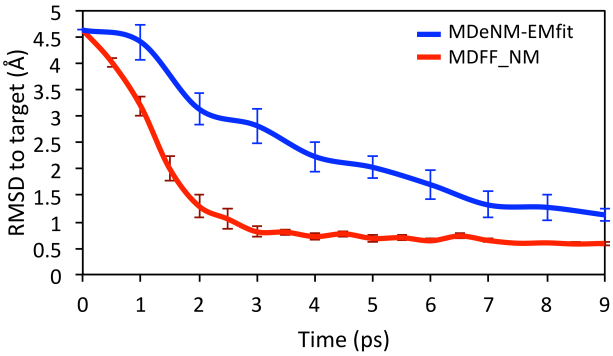

Supplementary Figure 1: Comparing MDFF_NM and MDeNM-EMfit in fitting simulations of LAO binding protein. Time evolution of the average RMSD taking as reference the 1LST structure considered for generating the target map at 5 Å resolution. Average values were computed from ten independent simulations performed in vacuum. Colored as indicated in the legend.

Supplementary Table 1: Comparison between Molprobity scores obtained for MDFF_NM solutions and their corresponding target states.

system	Source	Molprobity score
Adenylate Kinase	Target: 1AKE.pdb	2.56
	MDFF_NM solutions	2.3 ± 0.13
LAO *binding* protein	Target: 2LAO .pdb	2.23
	MDFF_NM solutions	2.4 ± 0.11

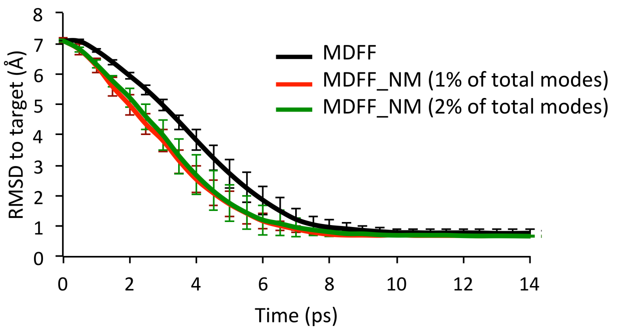

Supplementary Figure 2: Testing MDFF_NM parameters in flexible fitting simulations of adenylate kinase. Average RMSD with respect to the target structure considered for generating the 5 Å synthetic map (4AKE.pdb). For each evaluated approach, ten independent simulations in vacuum were performed. In addition, different percentages of normal modes defining the collective space were evaluated, as indicated in the legend.

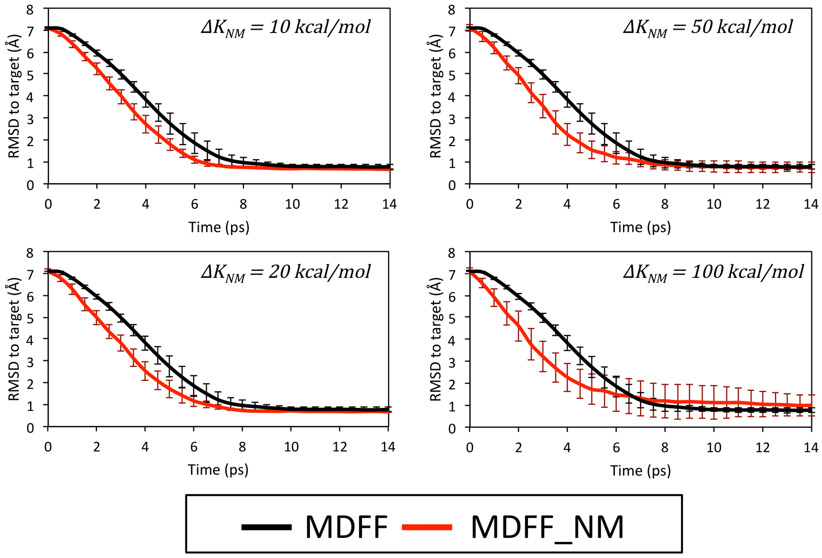

Supplementary Figure 3: Testing MDFF_NM parameters in flexible fitting simulations of adenylate kinase. Average RMSD with respect to the target structure considered for generating the 5 Å synthetic map (4AKE.pdb). For each evaluated approach, ten independent simulations in vacuum were performed. Different values of excitation energies injected along combinations of normal modes were considered (*ΔK_{NM}*), according to the legend in the top part of the plots.

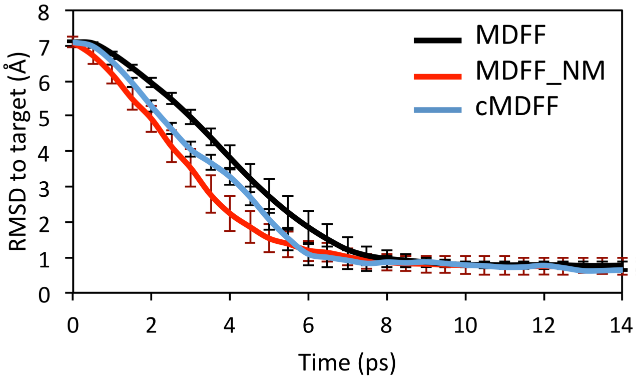

Supplementary Figure 4: Comparing different MDFF implementations in fitting simulations of adenylate kinase. Time evolution of the average RMSD taking as reference the 4AKE structure considered for generating the target map at 5 Å resolution. Average values were computed from ten independent simulations performed in vacuum. Colored as indicated in the legend.

List of PDB identifiers considered in the adenylate kinase ensemble

4ake	3x2s	5eje	6s36	4x8m
1e4v	6hap	4x8h	6ham	1ake
3hpr	6rze	1e4y		
